# Phase III study of 5FU, etoposide and leucovorin (FELV) compared to epirubicin, cisplatin and 5FU (ECF) in previously untreated patients with advanced biliary cancer

**DOI:** 10.1038/sj.bjc.6602576

**Published:** 2005-04-26

**Authors:** S Rao, D Cunningham, R E Hawkins, M E Hill, D Smith, F Daniel, P J Ross, J Oates, A R Norman

**Affiliations:** 1 Department of Medicine, Royal Marsden Hospital, Downs Road, Sutton, Surrey SM2 5PT, UK; 2 Department of Medicine, Royal Marsden Hospital, London, UK; 3 Christie Hospital, Manchester, UK; 4 Kent Oncology Centre, Maidstone, UK; 5 Clatterbridge Centre for Oncology, Clatterbridge, UK; 6 Plymouth Oncology Centre, Plymouth, UK

**Keywords:** chemotherapy, advanced biliary cancer, ECF, FELV, untreated phase III

## Abstract

The purpose of this study was to determine whether epirubicin, cisplatin and infused 5FU (ECF) improves overall survival (OS) compared to 5FU, etoposide and leucovorin (FELV) in patients with previously untreated advanced biliary cancer in a prospective randomised study. Patients were randomly assigned to receive epirubicin, cisplatin and infused 5FU ECF or bolus 5FU etoposide and leucovorin (FELV). The primary end point was OS with secondary end points of objective response rate (ORR), failure-free survival (FFS), quality of life (QOL) and toxicity. In all, 54 patients were recruited with 27 randomly assigned to each arm. The median OS for ECF was 9.02 months (95% confidence interval (CI): 6.46–11.51) and FELV 12.03 months (95% CI: 9.3–14.7), *P*=0.2059. Objective response rates were similar for both arms: ECF 19.2% (95% CI: 6.55–39.3); FELV 15% (95% CI: 3.2–37.9), *P*=0.72. There was significantly increased grade 3/4 neutropenia with FELV *vs* ECF (53.8 *vs* 29.5%, respectively, *P*=0.020). Symptom resolution was impressive for both regimens. This is the largest reported randomised study to date in this setting. ECF did not improve OS compared to FELV, but was associated with less acute toxicity. These data suggest that chemotherapy can prolong OS and achieve good symptomatic relief in advanced biliary cancer.

Biliary carcinoma is an uncommon malignancy, although its incidence appears to be increasing worldwide (Patel, 2001; Taylor-Robinson *et al*, 2001; Davila and El Serag, 2002; Khan *et al*, 2002). The incidence in the United States is approximately 8 per million and the majority of patients are over 65 years of age. The mortality rates of biliary cancer correspond to the incidence, as the prognosis is very poor. Surgery remains the only potentially curative intervention; however, the majority of patients present late with advanced unresectable disease.

Overall there are relatively few published data evaluating palliative chemotherapy for unresectable disease. Glimelius *et al* randomised patients with advanced biliary and pancreatic cancer to best supportive care plus or minus 5FU, etoposide and leucovorin (FELV). The median survival for the chemotherapy arm was significantly higher compared to best supportive care alone for all patients (6.5 *vs* 2.5 months, *P*<0.01), with a trend towards superior survival for the patients with biliary cancer (6.5 *vs* 2.5 months, *P*=0.10) (Glimelius *et al*, 1996). In addition, there was an improvement in overall quality of life (QOL) for those patients receiving chemotherapy. However, the chemotherapy group experienced considerable toxicity – 41% grades 3 and 4.

This was the only randomised trial reported in advanced biliary cancer at the time of designing our study. Several phase II studies had demonstrated activity with chemotherapy (both monotherapy and combination regimens), with response rates ranging from 0 to 34% (Harvey *et al*, 1984; Taal *et al*, 1993; Jones *et al*, 1996; Patt *et al*, 1996; Ducreux *et al*, 1998). We conducted a phase II study in this setting evaluating the combination of epirubicin, cisplatin and infused 5FU (ECF) based on encouraging activity and tolerance in oesophagogastric cancer. This demonstrated an objective response rate (ORR) of 40%, a median overall survival (OS) of 11 months and was associated with minimal grade 3/4 toxicity (Ellis *et al*, 1995).

The lack of randomised studies in this setting, the encouraging results produced by FELV chemotherapy *vs* best supportive care alone and the activity and tolerability of ECF in our phase II study led to the design of the randomised trial described in this report. The primary objective of this trial was to test whether ECF would improve the OS of patients with advanced biliary cancer compared to FELV chemotherapy.

## PATIENTS AND METHODS

### Study conduct

This multicentre randomised study was conducted in five centres in the United Kingdom. Signed informed consent was obtained from all patients prior to randomisation. The study was approved by the local institutional review boards at all participating centres.

### Main end points

Overall survival was the primary end point. The secondary end points were ORR, failure-free survival (FFS), toxicity and the impact of treatment on QOL.

### Patient selection

The main eligibility criteria were histologically or cytologically confirmed adenocarcinoma, squamous carcinoma or undifferentiated carcinoma of the gall bladder, intra/extrahepatic bile ducts or ampulla of Vater; no prior chemotherapy or radiotherapy; ECOG performance status (PS) 0–2; neutrophils >1.5 × 10^9^ l^−1^; platelets >100 × 10^9^ l^−1^; total white cell count >3.0 × 10^9^ l^−1^; total bilirubin <30 mmol l^−1^; glomerular filtration rate of >60 ml min^−1^; and life expectancy >3 months.

Patients were excluded if there were medical or psychiatric conditions precluding informed consent or significant cardiac disease, arrythmias or angina pectoris.

### Randomisation and study treatment

Eligible patients were centrally randomised electronically with stratification by centre. FELV comprised of 5FU 600 mg m^−2^ as intravenous (i.v.) bolus days 1–3; etoposide 120 mg m^−2^ i.v. infusion over 40 min days 1–3; and leucovorin 60 mg m^−2^ i.v. bolus days 1–3. Each cycle was repeated every 3 weeks.

ECF comprised of 5FU 200 mg m^−2^ by continuous infusion via a central line for 24 weeks; epirubicin 50 mg m^−2^ day 1; and cisplatin 60 mg m^−2^ with hydration day 1. Each cycle was repeated every 3 weeks.

### Dose modifications

Toxicity was evaluated and graded according to the National Cancer Institute common toxicity criteria (version 2.0). Any grade 3/4 nonhaematological toxicity resulted in treatment being withheld until recovery and then the following dose reductions were made: FELV 50% dose reduction in 5FU subsequently; ECF 50% dose reduction of infusional 5FU for grade 3 and 75% for grade 4. Any grade 3/4 haematological toxicity resulted in treatment being withheld until recovery and the following adjustments were made: FELV resumed at full dose for one delay, 25% dose reduction of 5FU and etoposide for 2 delays and 50% for more than 2 delays; ECF 25% dose reduction of epirubicin for grade 3 and 50% for grade 4 neutropenia, 50% for grade 3 and 75% for grade 4 thrombocytopenia.

### Safety evaluations

Patients were assessed at baseline with a full medical history and physical examination including PS, full blood count, serum biochemistry including electrolytes, hepatic and renal function. A baseline EDTA or 24 h urinary clearance was performed. During the study, full medical history and physical examination including PS, blood count, serum biochemistry including electrolytes, hepatic and renal function were performed prior to each cycle of treatment.

### Efficacy evaluations

Tumour response by CT assessment was performed according to WHO criteria at 12 and 24 weeks.

Overall survival and FFS were calculated for all randomised patients from the date of randomisation to the date of death or time to progression, respectively. Patients still alive were censored at the date of last contact.

### Quality of life

Quality of life was assessed with the European Organisation for Research and Treatment of Cancer (EORTC) QLQ-C30 questionnaire (incorporating five function scales, one global health-status scale and nine symptoms scales), which was filled in at baseline, 6, 12, 24 and 36 weeks.

### Statistics

The primary end point was OS and the study was designed to detect a 1-year survival of 43% for ECF compared to 20% for FELV. Thus, a total of 116 patients (58 per arm) were required based on a two-sided *α* of 5% (overall power=80%). Overall survival and FFS were compared between treatment groups using a two-sided log-rank test, and survival was calculated from the date of randomisation to the date of death or the date of last follow-up. Failure-free survival was calculated from the date of randomisation until the date of progression/death or the date of last follow-up. For each treatment, Kaplan–Meier estimates of median survival and its 95% confidence interval (CI) were computed. To adjust for confounding variables, multivariate Cox's regression models were used. Tumour response rates and toxicity in the two arms were compared using the *χ*^2^ test, and Fisher's exact test was used where appropriate. The change from baseline in QOL was compared between the two treatment arms using the Mann–Whitney test. *P*-values of less than 0.05 were considered to be statistically significant and all analyses were performed by intention to treat.

## RESULTS

Recruitment was slow and thus 27 patients were randomised to ECF and 27 to FELV between January 1997 and November 2003 in five centres in the United Kingdom. The patient baseline characteristics are shown in Table 1 and were generally well balanced between both groups, although there were more patients with bile duct carcinoma in the FELV group. The majority of patients were of PS 0–1.

### Treatment

The median duration of treatment was 5.5. cycles for ECF and six cycles for FELV. Dose reductions occurred in 18.5% of patients on ECF compared with 74% of patients receiving FELV. The main reason for dose reductions in the FELV arm was neutropenia. At least one dose delay occurred in 44.4% of patients in the ECF arm and 59.3% of patients in the FELV arm.

The dose intensity of each drug for both regimens is as follows: ECF epirubicin 88%; cisplatin 90%; 5FU 84%: FELV etoposide 89%; folinic acid 91.6%; and 5FU 76%. There was a trend towards a superior dose intensity of 5FU in the ECF cohort compared to FELV (84 *vs* 76%, *P*=0.056).

### Tumour response and symptomatic resolution

Eight patients were not evaluable for response: six had no radiologically measurable disease and two patients stopped treatment after only one cycle due to biliary stent infection and Hickman line infection, respectively.

Objective. response rates were similar for both arms (ECF 19.2% (95% CI: 6.6–39.3); FELV 15% (95% CI: 3.2–37.9), *P*=0.999 (Table 2)). A further proportion of patients in both arms achieved stable disease (ECF 46.2% compared to FELV 45%). The rate of progressive disease was comparable between both groups (ECF 34.6% *vs* FELV 40%).

Symptom resolution was achieved with both regimens ranging from 20 to 92% for a variety of symptoms including lethargy, pain, weight loss and anorexia (Table 3).

### Survival

**Figure 1:**
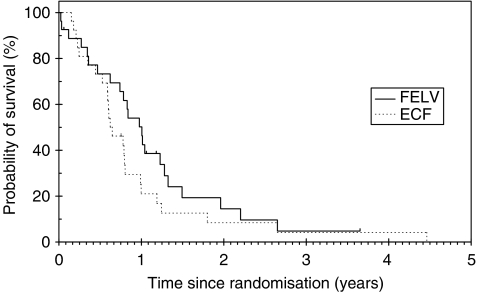
Overall survival (OS). With a median OS for ECF of 275 days (95% CI: 198–351) and 367 days for FELV (95% CI: 285–448), there was no statistically significant difference, *P*=0.2059.

With a median follow-up of 387 days, there was no statistically significant difference in median OS: ECF 9.02 mths. (95% CI: 6.46–11.51) and FELV12.03 mths (95% CI: 9.3–14.7), *P*=0.2059 (Figure 1). Multivariate analysis for OS confirmed these findings after controlling for performance status, locally advanced disease and alkaline phosphatase split on the median, there was no difference between arms, *P*=0.096. The 1 year OS for FELV was 50.2% (95% CI: 30–67.3) *vs* 21% (95% CI: 7.8–38.6) for ECF.

### Failure-free survival

**Figure 2:**
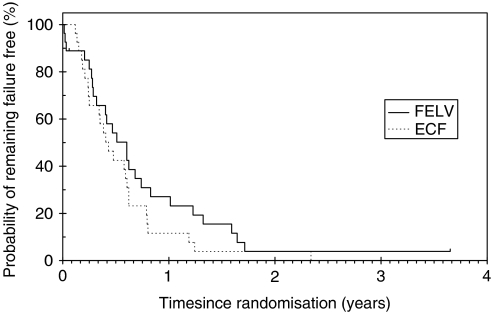
Failure-free survival (FFS). With a median FFS for ECF of 157 days (95% CI: 102.09–211.9) and for FELV 220 days (95% CI: 138–301.4), there was no statistically significant difference.

The median FFS for ECF was 157 days (95% CI: 102.09–211. 9) and for FELV 220 days (95%CI: 138–301.4). The 1 year FFS for FELV was 27.1% (95% CI: 12–44.7) *vs* 11.6% (95% CI: 2.9–26.8) for ECF (Figure 2).

### Quality of life

The compliance of patients in the QOL analysis was generally poor for both groups. The global QOL score at baseline for ECF and FELV: 62.9 *vs* 55.1 and at 12 weeks: 69.0 *vs* 70.83.

### Toxicity

The incidence of grade 3/4 adverse events is shown in Table 4. The non-haematological toxicity was similar between both groups aside from infection, which was significantly higher in the FELV arm. There was one treatment related death in the FELV arm due to sepsis and febrile neutropenia. There was a statistically higher incidence of grade 3/4 neutropenia for those patients receiving FELV compared to ECF (53.8 *vs* 29.5% respectively, *P*=0.02).

### Second line therapy

Four patients (18.5%). in the FELV arm went on to receive ECF on disease progression. 5 (18.5%) patients in the ECF group underwent further chemotherapy (2: carboplatin and 5FU; 1: 5FU and mitomycin C; 1: ZD 9331 and gemcitabine; 1 patient was re-challenged with ECF.

## DISCUSSION

At the time of designing this study there were relatively few published data for chemotherapy in advanced biliary cancer and thus a pragmatic approach was employed to evaluate the activity of ECF in this setting. The statistical design of the study was based on previously published data for ECF and FELV. Unfortunately accrual was very slow leading to closure after only 54 patients were enrolled over six years. The poor recruitment in our study is a reflection of this difficult population who often present at an advanced stage and are thus unfit for chemotherapy. Thus due to slow accrual the study was not adequately powered to detect a meaningful difference in survival between the two arms. Nevertheless this study remains the largest randomised trial in this setting to date.

ECF produced similar ORR, symptom resolution and FFS to FELV and was associated with significantly less acute toxicity. There was no difference in OS with FELV compared to ECF (median OS: 12.03 mths *vs* 9.02 mths HR 1.43 (95% CI: 0.81–2.84), *P*=0.2059).

This may be partly explained by 4 (18.5%) patients in the FELV arm receiving second line chemotherapy with ECF on disease progression. However 5 (18.5%) patients in the ECF group underwent further chemotherapy (2: carboplatin and 5FU; 1: 5FU and mitomycin C; 1: ZD 9331 and gemcitabine; 1 patient was rechallenged with ECF) therefore this is unlikely to have accounted for such a difference.

A potential criticism of this study design is the absence of an observational control arm. There was a survival benefit for all patients (biliary and pancreatic cancer) receiving FELV chemotherapy *vs* best supportive care in the Scandinavian randomised study (Glimelius *et al*, 1996). This difference was not significant when the biliary subset was analysed separately. However the study was only powered to detect a 6 months difference in OS with chemotherapy for all patients. Furthermore QOL was significantly improved for the chemotherapy subset, thus on balance we felt it reasonable to utilise FELV chemotherapy as the reference comparator arm in our trial.

Overall non-haematological toxicity was similar for both regimens. However there was significantly higher-grade 3/4 neutropenia and infection reported in the FELV arm. This resulted in sepsis and subsequent treatment related death in one patient. Myelosuppression was the main reason for treatment interruptions in the FELV group, this is reflected by the lower dose intensity of 5FU with this regimen compared to ECF (76 *vs* 84%, *P*=0.056).

Quality of life data for all patients was generally sparse due to poor attrition. Thus it is difficult to draw any meaningful conclusions. There was no statistically significant difference between global QOL scores over time for both arms. Symptom resolution was impressive and comparable in both regimens particularly for pain, weight loss and nausea. This is encouraging for this group of patients who frequently present with multiple symptoms.

There have been 2 other randomised studies reported for advanced biliary cancer. The Scandinavian study of 37 patients demonstrated a median OS of 6 months for FELV chemotherapy compared to 2.5 months for best supportive care (Glimelius *et al*, 1996). An EORTC study of 53 patients compared high dose 5FU with or without cisplatin. The median OS for 5FU alone was 5.3 months compared to 7.8 months for the combination arm (Mitry *et al*, 2002). In our study of 54 patients the median OS for FELV was 12.03 months and 9.03 months for ECF. Thus our data are encouraging and provide supporting evidence that chemotherapy can prolong OS in this disease.

A variety of chemotherapeutic agents have been investigated in this setting mainly in the context of phase II trials. Gemcitabine is of particular interest based on its significant activity in pancreatic cancer (Burris *et al*, 1997). It has been used as monotherapy and in combination with capecitabine, irinotecan, cisplatin and 5FU among others. Objective response rates range from 8–39% and tolerance is generally acceptable. (Kubicka *et al*, 2001; Penz *et al*, 2001; Jani *et al*, 2002; Jacobson *et al*, 2003; Thongprasert *et al*, 2003; Knox *et al*, 2004). Thus further randomised studies are underway or planned with these combination regimens *vs* gemcitabine alone.

In conclusion ECF produced similar ORRs, FFS and symptom resolution to FELV with less acute toxicity. As a result of poor recruitment the study was underpowered to detect a significant difference in OS between the two regimens. Thus based on these data it is not possible to define a reference regimen for advanced biliary cancer. Nevertheless this study has shown that chemotherapy can prolong OS and provide good symptomatic relief for these patients. Further adequately powered randomised trials are required to establish the standard of care for this disease.

## Figures and Tables

**Table 1:** Demographics

	**ECF (*N*=27)**	**FELV (*N*=27)**
Median age (years)	57	57
Age range (years)	36–70	39–76
Male (%)	40.7	59.3
PS 0–1 (%)	74.1	85.2
Metastatic	16 (59.3%)	18 (66.7%)
		
*Subsite*		
Ampulla	6 (22.2%)	4 (14.8%)
Gall bladder	14 (51.9%)	12 (44.4%)
Bile duct	7 (25.9%)	11 (40.7%)
		
*No. of disease sites*		
0–1	13	15
⩾2	14	12

ECF=epirubicin, cisplatin and 5FU; FELV=5FU, etoposide and leucovorin; PS=performance status.

**Table 2:** Objective response

	**ECF, *N*=26 (%)**	**FELV, *N*=20 (%)**
CR	1 (3.8)	—
PR	4 (15.4)	3 (15)
SD	12 (46.2)	9 (45)
PD	9 (34.6)	8 (40)

ECF=epirubicin, cisplatin and 5FU; FELV=5FU, etoposide and leucovorin; CR=complete response; PR=partial response; SD=stable disease; PD=progressive disease.

**Table 3:** Symptom resolution

**Symptom**	**ECF, *N* (%)**	**FELV, *N* (%)**
Pain	12/15 80	14/18 78
Anorexia	11/12 92	7/11 64
Weight loss	9/11 82	7/9 78
Nausea	2/3 67	3/4 75
Lethargy	6/14 43	3/15 20

ECF=epirubicin, cisplatin and 5FU; FELV=5FU, etoposide and leucovorin.

**Table 4:** Toxicity

	**ECF, *N*=25 (%)**	**FELV, *N*=24 (%)**
Diarrhoea	3 (12)	3 (12.5)
Stomatitis	2 (8)	2 (8.33)
Nausea and vomiting	4 (16)	2 (8.33)
Alopecia	10 (40)	18 (75)
Infection	4 (16)	10 (41.7)
Fever	3 (12)	2 (8.33)
Lethargy	14 (56)	14 (58.3)
Febrile neutropenia		1 (4.2)
		

	***N*=27 (%)**	***N*=26 (%)**
Anaemia	7 (25.9)	3 (11.5)
Thrombocytopenia	1 (3.7)	2 (8.3)
Neutropenia	7 (25.9)	14 (53.8)

ECF=epirubicin, cisplatin and 5FU; FELV=5FU, etoposide and leucovorin.
